# Structural integrity vs. clinical utility: a critical review of bio-inductive scaffolds and autologous alternatives in rotator cuff repair

**DOI:** 10.3389/fmed.2026.1826852

**Published:** 2026-06-17

**Authors:** Pingwen Lan, Zhi Fang, Bi Wu, Jianjun Zhang

**Affiliations:** Department of Orthopedics, People's Hospital of Deyang City, Deyang, China

**Keywords:** artificial intelligence, autologous augmentation, bio-inductive scaffold, health economics, precision medicine, retear, rotator cuff repair, tendon-to-bone healing

## Abstract

Despite major advances in arthroscopic fixation constructs, healing after large-to-massive rotator cuff repair remains limited by a persistent biological bottleneck: failure to regenerate the native graded tendon-to-bone enthesis. Bio-inductive collagen scaffolds have emerged as a translational strategy intended to enhance host-cell infiltration, angiogenesis, collagen deposition, and tendon-like tissue formation rather than simply bridging a structural defect. This critical review synthesizes current Level I–IV evidence from 2004 to 2026 regarding the biological rationale, structural efficacy, clinical translation, safety, economic value, autologous alternatives, and future precision-medicine applications of bio-inductive augmentation in rotator cuff repair. High-level evidence increasingly supports the ability of scaffold-based augmentation to improve structural integrity, with recent meta-analyses and randomized trials demonstrating lower retear rates and improved imaging-based healing in selected cohorts. However, a recurring biology-function gap remains evident: statistically significant improvements in tendon integrity do not consistently translate into patient-perceived gains exceeding established thresholds such as the minimal clinically important difference, patient acceptable symptom state, or substantial clinical benefit. This discrepancy likely reflects the multifactorial nature of postoperative recovery, including tear chronicity, muscle fatty infiltration, tendon quality, patient age, rehabilitation, and baseline functional status. Importantly, value-based caution should not be interpreted as a recommendation against bio-inductive implants in small-to-medium tears. Recent guideline-supported and randomized evidence indicates that selected small-to-medium lesions, particularly those with intact rotator cable integrity, compromised tendon quality, biological risk factors, or high return-to-work demands, may benefit from bio-inductive strategies. Conversely, indiscriminate use in low-risk tears with favorable healing potential remains difficult to justify, especially in the context of implant cost and emerging autologous alternatives such as long head of the biceps and fascia lata grafts. Future progress will depend on phenotype-specific indications, cost-effectiveness analyses, AI-assisted risk prediction, and next-generation gradient or bioactive scaffolds capable of more closely reproducing the native enthesis. Overall, bio-inductive scaffolds should be viewed not as universally indicated implants, but as selective biological tools whose clinical and economic value depends on matching mechanism, patient phenotype, and surgical objective.

## 1 Introduction: the epidemiological and biological imperative

The burden of rotator cuff pathology on the aging population is both substantial and escalating, constituting a primary source of shoulder dysfunction and pain. Population-based studies consistently demonstrate a strong age-dependent increase in rotator cuff tear prevalence, while epidemiological analyses of surgical utilization show a parallel rise in the number of rotator cuff repairs performed over time (1–3). Although many degenerative tears are initially asymptomatic, their natural history is frequently progressive rather than static. Prospective observational studies have shown that untreated tears may enlarge, become symptomatic, and develop irreversible muscle degeneration over time (4, 5). This biological progression is clinically important because fatty infiltration and muscle atrophy may not reliably reverse after repair and are associated with inferior functional outcomes (6). Thus, the clinical imperative in rotator cuff disease is not merely to close a tendon defect, but to intervene before a reparable tendon lesion evolves into an irreparable muscle-tendon unit disorder.

But does surgical repair reliably reverse this pathology? Despite the widespread adoption of arthroscopic techniques, the management of large-to-massive tears remains a persistent challenge in modern orthopedics. Historically, Galatz et al. (7) documented a sobering 94% retear rate in massive tears, a pivotal finding that ignited a two-decade quest for improved fixation strategies. Yet, this mechanical evolution, transitioning from simple single-row constructs to sophisticated transosseous-equivalent suture bridges, has arguably reached a point of diminishing returns. Contemporary evidence-based literature underscores that the risk of structural failure is not uniform but highly dependent on both tear size and biological host factors. Reviews and meta-analyses have shown that larger tear size, older age, tendon retraction, poorer muscle quality, and systemic risk factors are associated with impaired healing and recurrent structural failure (8–10). While small and medium tears generally exhibit more favorable healing profiles, large tears carry substantially higher retear risks, and massive or irreparable tears remain particularly vulnerable to failure despite modern fixation constructs (8–10). This size- and biology-dependent stagnation suggests that the limiting factor in large-to-massive tears is no longer simply the tensile strength of the suture-anchor construct, but rather the unyielding biology of tendon-to-bone healing.

The biological basis for this failure lies in the specialized architecture of the native enthesis. The healthy tendon-to-bone insertion is a graded transition zone composed of tendon, unmineralized fibrocartilage, mineralized fibrocartilage, and bone, allowing stress to dissipate gradually across tissues with markedly different stiffness (11). After surgical repair, however, this complex zonal architecture does not regenerate. Healing occurs predominantly through fibrovascular scar formation, which has inferior mechanical properties and creates a stress concentration at the repair interface (12). Consequently, as articulated in a recent editorial by Saithna, the field faces a biological bottleneck in which further optimization of suture configuration alone is unlikely to eliminate recurrent failure (13).

The recently released 2025 American Academy of Orthopaedic Surgeons (AAOS) Clinical Practice Guideline provides a robust evidence-based framework for managing rotator cuff injuries (14). However, while clinical guidelines offer essential recommendations on treatment selection, there remains a critical need for a deeper synthesis of the biological mechanisms, risk phenotypes, comparative biologic options, and health-economic implications that determine whether advanced augmentation technologies are truly justified in specific patients.

In response to this impasse, the treatment paradigm is shifting from “mechanical reinforcement” using thick, load-bearing grafts to a strategy of “biological induction.” Bio-inductive collagen implants (BCI) have emerged as a highly porous scaffold technology designed not primarily to bridge a defect mechanically, but to facilitate host-cell infiltration, neovascularization, extracellular matrix deposition, and increased tendon thickness (15–17). Human biopsy and imaging studies support the concept that these implants can induce a neotendon-like tissue layer that integrates with native tendon (16, 17). At the same time, contemporary reviews emphasize that scaffold-based augmentation, structural allograft reinforcement, injectable biologics, and autologous tissue augmentation should not be treated as interchangeable strategies, because each addresses a different failure mechanism and carries distinct biological, technical, and economic implications (18, 19). These findings position bio-inductive scaffolds as a promising but indication-sensitive solution to the regenerative deficiencies of the aging rotator cuff, and they also frame the central question of this review: whether improved structural integrity reliably translates into clinically meaningful utility and health-system value.

## 2 Biological rationale and preclinical evidence

### 2.1 The enthesis challenge: regeneration vs. repair

To fully appreciate the necessity of biological augmentation, one must first dissect the regenerative limitations of the native enthesis. As elucidated in the foundational work by Thomopoulos et al., the healthy tendon-to-bone insertion is not a discrete junction but a sophisticated, graded interface organized into four distinct zones: tendon, unmineralized fibrocartilage, mineralized fibrocartilage, and bone (11). This architecture is increasingly understood as a structure–function continuum rather than a simple anatomical boundary. Compositional and biomechanical studies have shown that collagen orientation, mineral content, and elastic modulus change gradually across the attachment site, thereby reducing abrupt stress concentrations between the compliant tendon and the rigid bone (20, 21). This gradient serves a critical biomechanical function: it effectively dissipates stress concentrations by allowing a gradual transition in stiffness from soft tissue to rigid bone.

**Figure 1:**
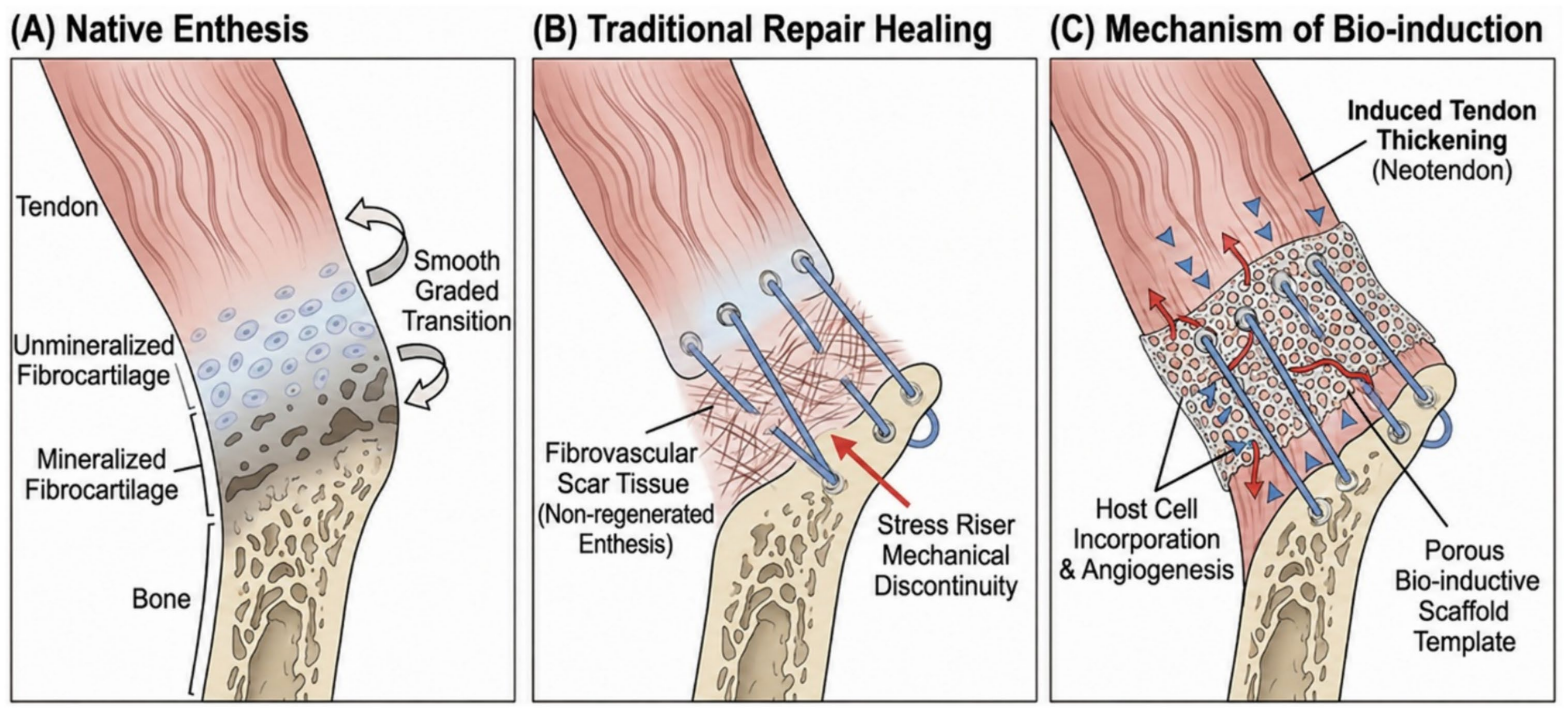
The biological challenge of rotator cuff healing and the mechanism of bio-inductive scaffolds. **(A)** Native enthesis: The healthy tendon-to-bone insertion is organized into a graded transition from tendon to unmineralized fibrocartilage, mineralized fibrocartilage, and bone. This structure–function continuum gradually changes in collagen organization, mineral content, and stiffness, thereby dissipating stress across the attachment site (11, 20, 21). **(B)** Traditional repair healing: Standard repair does not regenerate the native enthesis. Instead, healing occurs through fibrovascular scar tissue at the tendon-bone interface, which lacks the native fibrocartilage organization and mineral gradient required for durable load transfer, creating a potential mechanical “stress riser” (12, 22, 23). **(C)** Mechanism of bio-induction: The bio-inductive scaffold functions as a transient porous extracellular-matrix template rather than a permanent mechanical bridge. It supports host-cell infiltration, angiogenesis, collagen deposition, and tendon-like tissue maturation, and is gradually replaced by newly formed collagenous tissue that may increase tendon thickness and improve the local biological environment (16, 17, 24–26).

However, surgical repair fails to recapitulate this complex architecture. Instead of regenerating the native zonal gradient, healing occurs via the formation of a reactive, homogenous scar tissue interface. As Derwin et al. (12) highlight, this scar tissue possesses inferior mechanical properties and acts as a significant stress riser, predisposing the construct to failure at physiological loads far below the threshold of the native tendon. This limitation is also supported by broader tendon-to-bone healing literature showing that the repaired interface often lacks native fibrocartilage organization, mineral gradation, and mature Sharpey-like fiber continuity, all of which are required for durable load transfer (22, 23). This “regenerative gap” explains why mechanical fixation alone, regardless of anchor strength, often succumbs to biological failure (Figures 1A,B).

### 2.2 Mechanism of bio-induction: insights from animal models

The bio-inductive collagen implant was engineered specifically to bridge this regenerative deficit. In contrast to traditional synthetic scaffolds that rely on chemical cross-linking for mechanical stiffness, this device utilizes a highly porous, reconstituted bovine collagen architecture designed to be rapidly solubilized. Preclinical histological work in a sheep rotator cuff model demonstrated that reconstituted collagen scaffolds can support host tissue ingrowth and progressive replacement by newly formed tendon-like tissue, providing an important biological rationale for their clinical translation (24). Recent histological validation is provided by the systematic review of Longo et al. (17), which confirmed that the implant facilitates the rapid infiltration of fibroblasts and neovascular channels (Figure 1C).

The proposed mechanism is therefore not simple mechanical coverage of the tear surface. Rather, the porous collagen matrix functions as a transient extracellular-matrix template that absorbs local blood, marrow-derived elements, and synovial fluid, retains endogenous biological mediators at the tendon surface, and provides a scaffold for fibroblast migration, angiogenesis, collagen deposition, and tissue maturation. Human biopsy evidence has demonstrated organized, tendon-like collagen formation after implantation (16), and prospective imaging studies of partial-thickness tears have reported increased tendon thickness and progressive defect filling after collagen implant augmentation (25, 26). Together, these preclinical, histological, and imaging data strengthen the biological premise that bio-inductive implants act primarily by modulating the host healing response rather than by serving as a permanent structural substitute.

### 2.3 Biomechanical nuance: induction, not fixation

It is clinically paramount to distinguish the biomechanical function of these bio-inductive scaffolds from that of structural allografts (e.g., human dermal matrices). Structural patches are load-bearing devices intended to mechanically bridge a defect at “time zero.” Conversely, bio-inductive implants provide negligible initial mechanical strength. They rely entirely on the biological induction of new tissue over time to share load. This distinction is consistent with contemporary systematic reviews that separate scaffold-based induction from graft-based structural reinforcement, emphasizing that these technologies address different failure mechanisms and should not be considered interchangeable (18, 19). This distinction is supported by human biopsy studies from Arnoczky et al. (16), which confirmed that the “neotendon” formed in patients is histologically indistinguishable from native tendon, organizing into parallel collagen bundles that mature and integrate over a 6-month period. Thus, the efficacy of this technology rests not on immediate reinforcement, but on the successful biological modulation of the healing environment.

This biomechanical nuance has direct implications for patient selection. In repairable tears with poor tendon biology but adequate residual tissue, a bio-inductive scaffold may enhance the biological quality and thickness of the repaired tendon. In contrast, when a tear is irreparable or requires gap bridging, a scaffold with minimal initial tensile strength should not be expected to replace a structural graft or reconstructive procedure. The core indication is therefore biological augmentation of a repairable tendon bed, not primary mechanical reconstruction of an unreconstructable defect.

## 3 Comparative biologics: positioning in the treatment algorithm

The integration of biologics into rotator cuff repair is not a monolithic endeavor; rather, it necessitates a nuanced selection between structural reinforcements (e.g., human dermal allografts), bio-inductive scaffolds (e.g., bovine collagen implants), and injectable biologics (e.g., PRP). Each modality targets a specific failure mechanism, yet their optimal indications often overlap, creating a decision-making dilemma for the surgeon. Contemporary reviews increasingly emphasize that these biologic strategies should be categorized according to their dominant therapeutic function: mechanical load-sharing, host-tissue induction, or transient molecular signaling (18, 19). This distinction is clinically important because grafts, scaffolds, and injectables differ substantially in time-zero biomechanics, degradation kinetics, host incorporation, technical complexity, and cost. Treating these modalities as interchangeable risks obscuring both their biological rationale and their most appropriate clinical indications.

### 3.1 Structural allografts vs. bio-inductive scaffolds: a philosophical shift

Historically, human dermal allografts (HDA) served as the cornerstone of augmentation strategies. As delineated in the comprehensive systematic review by Mandalia et al. (18), these chemically cross-linked matrices are engineered to provide immediate load-sharing competence at time zero, effectively shielding the repair from early mechanical failure. The clinical rationale for structural allograft augmentation is strongest in large, massive, revision, or poor-tissue-quality tears in which native tendon coverage and mechanical competence are insufficient. Early randomized evidence by Barber et al. (27) showed improved repair integrity after acellular human dermal matrix augmentation compared with repair alone, supporting the concept that structural patches can improve tendon healing in selected high-risk repairs. Subsequent systematic reviews have similarly suggested that graft augmentation may reduce retear rates in large-to-massive tears, although the strength of inference is limited by heterogeneity in graft material, tear chronicity, fixation strategy, imaging definitions, and follow-up duration (18, 28). However, this structural rigidity introduces a “double-edged sword”: the potential for stress shielding, which may inhibit native tendon remodeling, and the technical complexity of suturing a thick graft in a tight subacromial space.

In stark contrast, bio-inductive scaffolds represent a philosophical pivot from “replacement” to “induction.” Devoid of significant initial tensile strength, they function instead as a highly porous template for host tissue generation. This distinction is supported by histological and imaging evidence showing fibroblast infiltration, neovascularization, collagen deposition, and increased tendon thickness after bio-inductive collagen implant placement (16, 17, 25, 26). Systematic reviews of bio-inductive collagen implants further suggest favorable structural and MRI outcomes, while also emphasizing that clinical superiority remains indication-dependent and not uniform across all tear patterns (17–19). While Mandalia’s meta-analysis confirms that both modalities effectively reduce retear rates relative to standard repair, bio-inductive implants are increasingly favored for their lower profile and streamlined application, particularly in partial-thickness defects where a bulky dermal patch might provoke subacromial impingement (18). Thus, the key clinical distinction is not whether one biologic is universally superior, but whether the dominant failure mode is mechanical insufficiency requiring load-sharing graft reconstruction or biological insufficiency in a repairable tendon bed that may benefit from scaffold-mediated tissue induction.

### 3.2 The scaffold advantage over injectables (PRP)

This leads to a pivotal question. If the objective is biological signaling, why incur the morbidity of implanting a scaffold when one could simply inject platelet-rich plasma (PRP)? The current PRP literature provides a useful comparator because meta-analyses suggest that PRP may reduce retear rates after rotator cuff repair, but its effects on pain, function, and patient-reported outcomes are inconsistent and often modest (29, 30). These findings indicate that transient biologic signaling alone may be insufficient to reliably overcome the hostile tendon-to-bone healing environment in high-risk repairs.

The answer lies in retention. While PRP delivers a potent bolus of growth factors, it lacks a physical matrix to sustain their presence at the healing site. A 2025 review by Weiss et al. (31) identifies the “washout effect” inherent to the fluid dynamics of arthroscopy as a critical limitation of injectables, leading to the rapid dispersion of bioactive factors. Herein lies the distinct advantage of bio-inductive scaffolds: by providing a stable, highly porous collagen network, they act as a biological sponge. This architecture retains endogenous or exogenous signaling molecules directly at the footprint, suggesting that the scaffold’s primary value may not be the collagen itself, but its function as a durable delivery vehicle that outlasts the transient effects of single-shot injections (31). Accordingly, PRP may be best conceptualized as a short-duration molecular adjunct, whereas bio-inductive scaffolds provide both a local retention platform and a three-dimensional extracellular-matrix template for sustained host-cell recruitment and matrix deposition. This framework helps explain why scaffold-based augmentation may be more suitable for biologically compromised tendon beds, while injectable biologics may be more appropriate as adjunctive signaling therapies in lower-risk repairs or when a physical implant is not warranted.

Taken together, comparative biologic selection should be mechanism-driven rather than technology-driven. Structural allografts are most rational when mechanical load sharing or gap bridging is required; bio-inductive scaffolds are most rational when the tendon is repairable but biologically compromised; and PRP is most rational when transient signaling enhancement is desired without introducing an implant. This mechanism-based framework provides the foundation for the stratified treatment algorithm discussed later in this review.

## 4 Surgical considerations and technical evolution

### 4.1 Fixation technology: a paradigm shift from sutures to Staples

The widespread clinical assimilation of bio-inductive scaffolds has been significantly accelerated by innovations in delivery systems that minimize technical complexity. Unlike structural allografts, which typically necessitate labor-intensive suturing to the remnant tendon, a process that not only prolongs operative time but also increases the risk of fluid extravasation, modern bovine collagen implants employ a streamlined fixation strategy using proprietary poly-lactic-co-glycolic acid (PLGA) staples. Prospective multicenter studies and early safety series have described this low-profile, bursal-sided deployment strategy as technically reproducible, with rapid fixation and favorable short- to mid-term structural outcomes when the scaffold is used to augment repairable full-thickness or high-risk tears (15, 32, 33). This efficiency offers a dual benefit: it reduces the physiological burden on the patient by shortening anesthesia time and mitigates the “technical fatigue” often associated with complex dermal allograft reconstruction.

The technical advantage of staple fixation should not be interpreted as a license for casual placement. Unlike dermal allografts, which are tensioned and sutured as load-sharing constructs, bio-inductive scaffolds require broad, wrinkle-free contact with the bursal tendon surface and the prepared vascular bed to maximize cell ingress and matrix deposition (25, 26, 32). Appropriate medial-lateral positioning, adequate overlap beyond the degenerative tendon margin, and avoidance of implant folding are therefore central to the biological success of the procedure. In comparative terms, the lower technical burden of bio-inductive scaffolds is strongest when the clinical goal is biological surface induction rather than mechanical gap bridging, whereas structural grafts remain more appropriate when time-zero load sharing is required (18, 27, 28).

Recent comparative clinical data also suggest that the procedural value of bio-inductive repair may include operative efficiency. For high-grade partial-thickness tears, Doyle et al. (34) reported comparable clinical outcomes between bio-inductive implant repair and tear-completion repair, while highlighting reduced operative time as a potential advantage of the implant-based strategy. This finding supports the concept that surgical efficiency, when paired with appropriate biological indications, may represent a legitimate component of scaffold utility rather than a purely logistical benefit.

### 4.2 Optimizing the biological bed: the “Crimson Duvet”

However, secure fixation alone is insufficient for integration; the host bed must be primed to receive the graft. Central to this biological preparation is the marrow-venting or “Crimson Duvet” concept. Microfenestration of the greater tuberosity footprint is intended to release marrow-derived cells and vascular channels into the repair site; this biological premise is supported by evidence that bone marrow-derived cells from the footprint can infiltrate the repaired rotator cuff, and by clinical scaffold protocols emphasizing biological footprint preparation (33, 35). This technique involves extensive micro-fenestration of the greater tuberosity footprint, a maneuver designed not merely to induce bleeding, but to actively recruit bone marrow-derived mesenchymal stem cells (MSCs) and growth factors to the repair site. The biological rationale for marrow stimulation is supported by randomized clinical evidence showing that marrow-stimulating techniques can improve tendon integrity after arthroscopic rotator cuff repair in selected tears, although the magnitude of benefit may depend on tear size, tissue quality, repair configuration, and rehabilitation protocol (36). By permeating the highly porous scaffold with this bioactive milieu, the surgeon transforms an inert collagen sheet into a dynamic, regenerative environment, thereby maximizing the inductive potential of the matrix.

This point is particularly important because bio-inductive scaffolds are biologically permissive rather than biologically autonomous. Their performance depends on the availability of host cells, vascular channels, and endogenous signaling molecules within the prepared repair bed. Consequently, meticulous bursectomy, gentle debridement of nonviable tendon surface, controlled decortication or microfenestration of the footprint, and maintenance of scaffold-to-tendon contact should be regarded as integral components of the implant procedure rather than optional adjuncts (24–26, 33, 35, 36). Conversely, excessive footprint decortication may compromise anchor purchase or disrupt remaining native enthesis tissue, underscoring the need for balanced preparation rather than indiscriminate bleeding induction.

### 4.3 Technical pitfalls and the learning curve

Despite the intuitive nature of the delivery system, the technique is not devoid of complications, necessitating a nuanced understanding of implant biomechanics. Hurley et al. (19), in a systematic review of complications, cautioned that while rare, issues related to implant prominence or staple pull-out can occur, potentially leading to mechanical abrasion of the acromion or secondary chondral damage. Consequently, precise staple placement, ensuring fasteners are flush with the tendon surface and positioned medially enough to avoid subacromial impingement during abduction, is paramount. Furthermore, surgeons must navigate the learning curve associated with manipulating the hydrated, gelatinous scaffold, which requires delicate handling to prevent intraoperative fragmentation before fixation is achieved.

Several practical failure modes deserve explicit attention. Inadequate bursal clearance can obscure the tendon margins and lead to malposition; excessive lateral placement can increase the risk of subacromial abrasion; insufficient fixation may permit scaffold migration or edge lift-off; and overtensioning or folding of the implant may reduce effective contact area and impair host incorporation. These technical concerns are especially relevant in revision cases, poor-quality tendon, and narrow subacromial spaces, where visualization and working volume are limited (19, 32, 33).

Postoperative management should also reflect the implant’s biological rather than structural role. Because the scaffold contributes little immediate tensile strength, early rehabilitation should not be based on the assumption that the implant mechanically protects the repair. Instead, protocols should respect the underlying repair construct, tear size, tendon quality, and patient-specific risk profile, while avoiding unnecessary immobilization that may contribute to stiffness. This balanced approach is consistent with the broader literature showing that scaffold augmentation may improve the biological environment of healing but does not eliminate the need for standardized, indication-specific postoperative rehabilitation (15, 19, 32, 33).

## 5 The evidence for structural integrity

The principal metric for defining the efficacy of any biological augmentation strategy remains its ability to reverse the historical trajectory of recurrent structural failure. Recent Level I and systematic-review evidence has strengthened this structural argument. In a 2024 meta-analysis of randomized controlled trials, Hurley et al. (37) reported that acellular collagen matrix patch augmentation reduced the retear rate from 34.9% in non-augmented controls to 11.0% in augmented repairs (RR = 0.36; *p* = 0.0006). This protective effect is not an isolated observation; Orozco et al. (38) similarly found that patch augmentation was associated with significantly lower retear rates in large tears, with healing rates diverging between augmented and non-augmented groups (11.4% vs. 28.2%; *p* = 0.0001). Together, these data suggest that the most reproducible benefit of scaffold-based augmentation is structural rather than symptomatic.

Importantly, the structural signal is supported not only by pooled estimates but also by prospective and randomized clinical evidence. In the Level I trial by Ruiz Ibán et al. (39), augmentation of transosseous-equivalent repair with a bioinductive collagen implant significantly reduced retears at 1 year and, more specifically, mitigated Type 2 medial failures. In selected small/medium full-thickness supraspinatus tears with an intact rotator cable, Camacho Chacón et al. (40) reported complete MRI healing at 12 and 24 months after isolated bioinductive repair, along with increased tendon thickness and superior biopsy-derived tendon quality. Prospective multicenter and cohort studies have also reported progressive tendon thickening, favorable MRI appearance, and sustained repair integrity after bioinductive implant augmentation across partial- and full-thickness tear settings (15, 33).

These findings provide biological plausibility for the structural effect observed clinically. Histological and imaging-based systematic evidence indicates that bioinductive implants can support host-cell infiltration, vascular ingrowth, collagen deposition, and maturation of tendon-like tissue, rather than functioning as inert coverings (17). Thus, the observed reduction in retear rate is likely not attributable solely to a mechanical barrier effect; rather, it reflects a time-dependent biological process in which scaffold-guided tissue formation increases tendon thickness and may improve load distribution across the repaired footprint.

However, aggregate data can obscure the biomechanical nuance of how protection is achieved. The failure mode is clinically relevant: medial-row or Type 2 failures are particularly problematic after transosseous-equivalent repair because they occur near the musculotendinous junction, where tissue quality and strain concentration may be unfavorable. Ruiz Ibán et al. (39) provided important granularity by showing that bioinductive augmentation reduced Type 2 medial failures, supporting a “check-rein” or load-sharing hypothesis in which newly induced tissue protects the vulnerable medial repair zone. Complementing this concept, Krupp et al. (41) reported that an interpositional scaffold-anchor system designed to integrate the graft directly into the footprint achieved a 95.7% healing rate in moderate-to-large tears, suggesting that structural benefit may be reproducible across different scaffold delivery platforms.

Nevertheless, the structural literature should be interpreted with appropriate caution. Studies differ in tear size, chronicity, repair construct, scaffold type, imaging modality, follow-up duration, and definitions of healing or retear. Moreover, although structural integrity is a prerequisite for durable tendon restoration, it is not equivalent to clinical success. The current evidence therefore supports a focused conclusion: bioinductive and scaffold-based augmentation can reliably improve anatomical healing in selected rotator cuff repairs, particularly in structurally high-risk tears, but the magnitude of this radiographic benefit must still be weighed against patient-reported outcomes, cost, and phenotype-specific indications (15, 17, 27, 33, 37–41) (Table 1).

**Table 1:** Key evidence summary: structural integrity, clinical translation, and comparative augmentation strategies in rotator cuff repair.

Study	Level of evidence	Comparison groups/population	Structural outcome	Clinical outcome (PROs)	Key conclusion
Hurley et al. (37)	Level I (Meta-analysis of RCTs)	Acellular collagen matrix patch augmentation vs. non-augmented repair	Significant retear reduction (11.0% vs. 34.9%; RR = 0.36; *p* = 0.0006)	Statistical ASES improvement, but improvement did not clearly exceed MCID	Strongest pooled evidence that scaffold-based augmentation improves structural healing; clinical translation remains less consistent.
Orozco et al. (38)	Systematic review of RCTs	Patch augmentation vs. no patch, particularly in large tears	Lower retear rates favoring augmentation (11.4% vs. 28.2%; p = 0.0001)	No significant differences in Constant, ASES, or UCLA scores	Supports a reproducible structural benefit while reinforcing the biology-function gap.
Ruiz Ibán et al. (39)	Level I RCT	Bioinductive collagen implant + TOE repair vs. TOE repair alone in posterosuperior nonacute tears	Reduced 1-year retear rate, especially Type 2 medial failures	No significant between-group functional-score difference at 12 months	Provides failure-mode granularity and supports a medial-row protective or check-rein mechanism.
Camacho Chacón et al. (40)	Level I RCT	Isolated bioinductive repair vs. sutured repair in selected small/medium full-thickness supraspinatus tears with intact rotator cable	Superior biopsy tendon quality; increased tendon thickness; 100% MRI healing at 12 and 24 months	Higher ASES/CMS, less pain, greater satisfaction, and faster return to work	Supports isolated bioinductive repair as an effective alternative strategy in selected small/medium tear phenotypes.
Bushnell et al. (15)	Prospective multicenter study	Bioinductive collagen implant augmentation in full-thickness rotator cuff repair	Progressive tendon thickening and favorable MRI integrity at 2 years	Sustained clinical improvement from baseline	Provides multicenter clinical and imaging support for tendon-thickening and structural-healing effects.
Thon et al. (33)	Prospective clinical study	Bioinductive collagen patch for large and massive rotator cuff tears	Favorable healing/safety profile at 2-year follow-up	Improved clinical outcome scores from baseline	Extends bioinductive implant evidence into structurally high-risk large/massive tears.
Longo et al. (17)	Systematic review	Histological, clinical, and MRI outcomes after bioinductive collagen implant use	Evidence of tendon thickening, host-cell infiltration, vascular ingrowth, and tendon-like tissue formation	Clinical scores generally improved after treatment, though comparative superiority varied	Synthesizes biological plausibility for scaffold-guided tissue induction rather than simple mechanical coverage.
Krupp et al. (41)	Prospective cohort / device-platform study	Interpositional scaffold-anchor system in moderate-to-large footprint tears	95.7% healing rate reported	Early clinical improvement	Suggests structural benefit may be reproducible across different scaffold delivery platforms.
Barber et al. (27)	Prospective randomized trial	Acellular human dermal matrix augmentation vs. standard arthroscopic repair	Improved repair integrity in augmented repairs	Clinical improvement in both groups	Provides high-quality foundational evidence that biologic patch augmentation can improve structural integrity.
Doyle et al. (34)	Level III comparative study	Bioinductive implant repair vs. tear completion and repair for high-grade partial-thickness tears	Comparable structural outcomes	No significant functional difference at 1 year	Shows that in some lower-severity tears, utility may relate to surgical efficiency and recovery trajectory rather than superior final PROs.
Baumann et al. (51)	Level I (Systematic review/meta-analysis)	Partial repair with vs. without long-head-of-biceps augmentation for large-to-massive tears	Reduced retear rate in massive/irreparable tears (42.9% vs. 72.5%; *p* = 0.007)	Comparable or improved clinical outcomes	Supports autologous augmentation as a cost-conscious structural alternative in high-risk massive tears.

## 6 The clinical paradox: statistical vs. clinical significance

### 6.1 The “paper success” phenomenon

While the anatomical data compellingly supports the structural efficacy of bio-inductive scaffolds, the translation of this radiological success into tangible patient-reported benefit remains more complex than a binary healed-versus-failed construct. This distinction is central to outcome interpretation because statistical significance does not necessarily indicate that a patient perceives a meaningful improvement; the concept of the minimal clinically important difference (MCID) was developed specifically to separate measurable change from clinically meaningful change (42). In shoulder surgery, related thresholds such as the patient acceptable symptomatic state (PASS) and substantial clinical benefit (SCB) are particularly relevant because many patients improve substantially after rotator cuff repair regardless of whether small between-group differences can be detected statistically (43).

Within the bio-inductive scaffold literature, this issue is exemplified by the recent meta-analysis by Hurley et al. (37), which demonstrated a marked reduction in retear risk with scaffold augmentation but only limited between-group differences in patient-reported outcome measures that may not consistently exceed accepted MCID thresholds. Similarly, Orozco et al. (38) reported lower retear rates after patch augmentation but found no consistent superiority in Constant, ASES, or UCLA scores compared with non-augmented repair. These data do not negate the structural value of augmentation; rather, they indicate that improved tendon continuity may not automatically translate into proportionally greater pain relief, strength, or function in every clinical phenotype.

**Figure 2:**
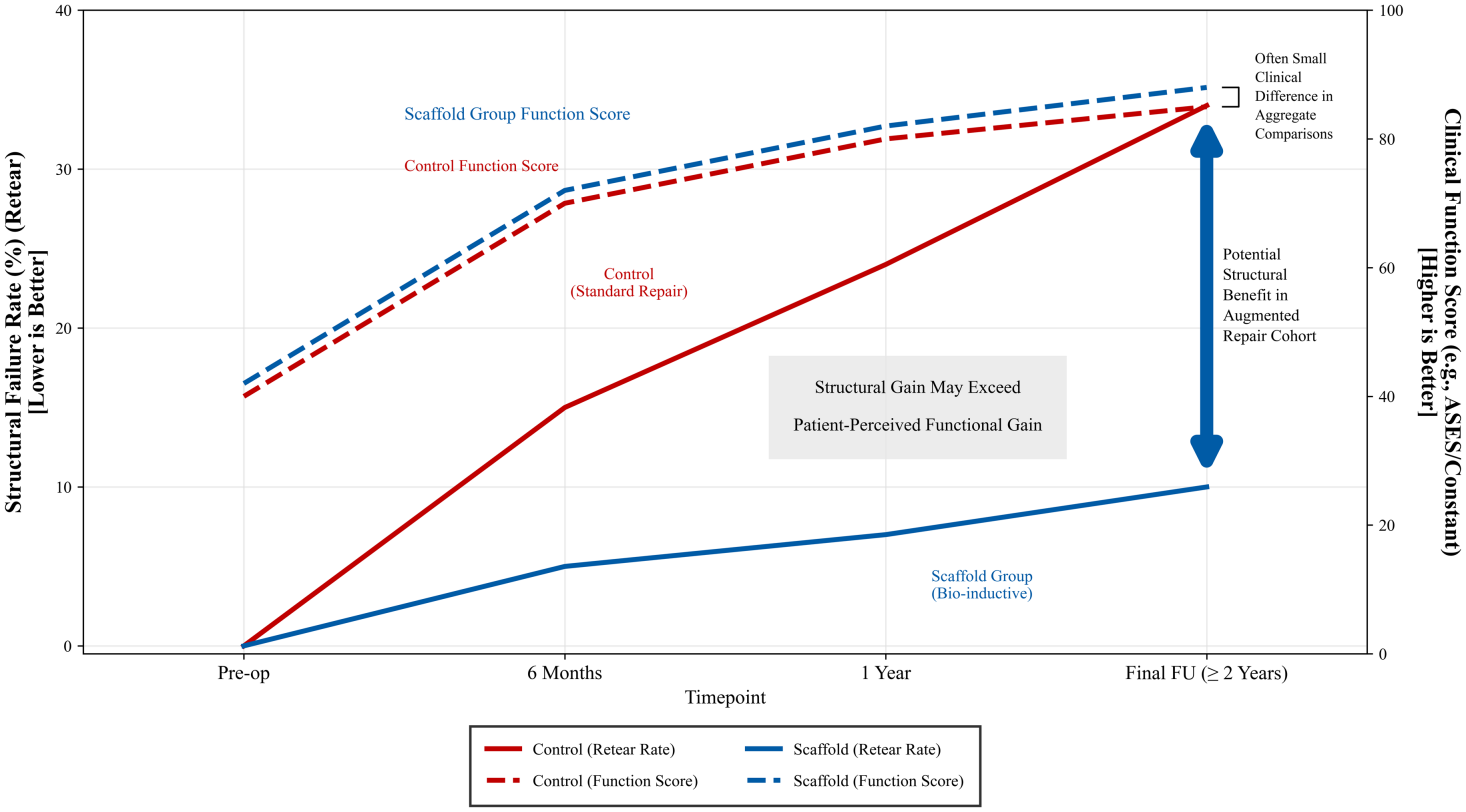
The “clinical paradox” in many augmentation studies: structural integrity does not always translate into proportionally greater patient-reported outcomes. This dual-axis conceptual plot illustrates a recurring pattern in bio-inductive or scaffold-based augmentation studies: structural healing may improve more substantially than conventional patient-reported outcome measures. Solid lines indicate structural failure, or retear rate, on the inverted left Y-axis, where lower values represent better healing. Several augmented repair cohorts and meta-analyses have reported lower retear rates compared with non-augmented repair, supporting a meaningful structural benefit (15, 17, 27, 33, 37–39, 41). Dashed lines indicate clinical function scores, such as ASES or Constant-Murley scores, on the right Y-axis. In some comparative studies, both groups improve substantially from baseline, but between-group differences in patient-reported outcomes remain small, non-significant, or below clinically meaningful thresholds such as MCID, PASS, or SCB (6, 34, 37–39, 42, 43). This biology-function gap suggests that improved anatomical continuity does not necessarily produce a proportional patient-perceived functional advantage. Functional recovery is also influenced by fatty infiltration, muscle atrophy, tear chronicity, biomechanics, rehabilitation, and patient-specific factors (5, 9, 27–30, 44–47). Therefore, this figure should be interpreted as a conceptual summary of many augmentation studies, not as evidence that bio-inductive implants lack clinical value in appropriately selected patients, including selected small-to-medium tears (9, 40).

Several mechanisms may explain this apparent biology-function gap. First, many patients experience meaningful pain relief after standard repair alone, producing a ceiling effect that makes additional gains difficult to detect using conventional PRO instruments (43). Second, patient-perceived function is influenced by factors beyond tendon integrity, including baseline pain sensitization, stiffness, deltoid and periscapular compensation, rehabilitation adherence, occupational demands, and psychological expectations. Third, chronic muscle changes may constrain functional recovery even when the tendon heals anatomically. Fatty infiltration and atrophy are known to correlate with poorer functional outcome and may not reliably reverse after repair (6). Thus, structural endpoints remain biologically important, but they should be interpreted alongside MCID, PASS, and phenotype-specific patient goals rather than as stand-alone surrogates for clinical success. This recurring divergence between structural healing and patient-perceived functional improvement is summarized conceptually in Figure 2.

### 6.2 Clinical neutrality, equivalence, and recovery trajectory

The paradox extends beyond meta-analyses into high-granularity comparative trials. In the Level I trial by Ruiz Ibán et al. (39), augmentation of a transosseous-equivalent repair with a bio-inductive collagen implant decreased structural failure, particularly Type 2 medial failures, yet functional scores at 12 months were not significantly different from the control group. This finding supports the concept that bio-inductive scaffolds may provide an anatomical or failure-mode advantage without necessarily producing a parallel short-term improvement in conventional PROs.

A similar pattern is observed in partial-thickness pathology. Doyle et al. (34) compared bio-inductive implant repair for high-grade partial-thickness tears with tear completion and repair and found comparable functional outcomes at one year, while the implant-based approach reduced operative time. This finding shifts the interpretation from simple superiority to clinical equivalence with potential workflow advantages. In selected small/medium full-thickness tears, however, the randomized trial by Camacho Chacón et al. (40) showed that isolated bio-inductive repair may improve not only healing and tendon quality but also patient-reported outcomes and return-to-work timing compared with sutured repair. Therefore, the clinical relevance of bio-inductive technology appears to depend strongly on indication, comparator strategy, and patient phenotype rather than on scaffold use alone (Table 1).

Accordingly, the utility of bio-inductive collagen implant repair should be assessed across multiple dimensions: final PRO scores, structural healing, early pain relief, rehabilitation progression, operative time, time to return to work, and risk of subsequent failure. Prospective multicenter data and systematic reviews have consistently shown tendon-thickening and MRI healing signals after bio-inductive implantation (15, 17), but these benefits should be weighed against the possibility that some cohorts may achieve similar final PROs after standard repair. This multidimensional interpretation provides a more rigorous framework than treating function scores alone as the sole determinant of value.

### 6.3 Reconciling guideline-supported small/medium tear indications with value-based caution

The 2025 American Academy of Orthopaedic Surgeons Clinical Practice Guideline provides a strong recommendation, based on high-quality evidence, that bioinductive tendon implants used either to augment rotator cuff repair or as an alternative to non-augmented repair can lead to lower retear rates and improved patient-reported outcomes (9). This recommendation is particularly relevant to the randomized controlled trial by Camacho Chacón et al. (40), which evaluated isolated bioinductive repair versus sutured repair in small/medium full-thickness supraspinatus tears with an intact rotator cable. In that selected phenotype, isolated bioinductive repair demonstrated superior tendon quality on biopsy, increased tendon thickness, complete MRI healing at 12 and 24 months, improved ASES and Constant-Murley scores, greater patient satisfaction, and faster return to work compared with sutured repair (40).

Therefore, small-to-medium tear size alone should not be interpreted as an exclusion criterion for bio-inductive technology. The apparent discrepancy between guideline-supported indications and the value-based caution emphasized in this review reflects differences in patient selection, tear phenotype, and comparator strategy. Camacho Chacón et al. (40) evaluated isolated bio-inductive repair as an alternative surgical strategy in a carefully selected small/medium tear population. By contrast, the caution raised in this review is directed toward the indiscriminate addition of costly commercial implants to all biologically low-risk tears, particularly when standard repair already has a high likelihood of structural healing and satisfactory clinical recovery (34, 37, 38).

This phenotype-specific approach is supported by a broader prognostic literature showing that rotator cuff healing is influenced by multiple patient- and tear-related factors rather than tear size alone. In small-to-medium tears, Park et al. (44) reported that infraspinatus fatty degeneration, anteroposterior tear size, and patient age were significant predictors of structural healing after arthroscopic repair. The Rotator Cuff Healing Index further formalized this concept by integrating tendon retraction, infraspinatus fatty infiltration, anteroposterior tear size, age, bone mineral density, and work activity into a multidimensional prediction tool (45). Large clinical datasets similarly show that increasing age and tear size reduce healing rates after repair (9), while meta-analytic evidence supports fatty infiltration as an important risk factor for retear and inferior functional recovery (46).

These findings reinforce the concept that small-to-medium tears are biologically heterogeneous. A small or medium tear with preserved tendon quality, minimal retraction, low-grade fatty infiltration, younger age, favorable bone quality, and low functional demand may have a high likelihood of healing with standard repair alone. Conversely, a tear of similar size but with poor tissue quality, advanced fatty infiltration, older age, reduced bone quality, metabolic risk factors, or high occupational demand may represent a substantially different healing phenotype and may justify consideration of a bio-inductive strategy (9, 44–46).

Accordingly, our position should be understood as advocating selective, phenotype-specific use rather than recommending against bio-inductive augmentation in small-to-medium lesions. Bio-inductive implants may be appropriate when tear morphology, tendon quality, rotator cable integrity, patient-specific biological risk factors, or return-to-work demands favor a bio-inductive approach (9, 40). Conversely, in low-risk tears with robust tendon quality, minimal retraction, preserved muscle quality, and low functional demand, the incremental value of routine implant use should still be weighed against cost, operative workflow, and expected baseline healing potential (34, 37, 38).

### 6.4 Mechanisms behind the biology-function gap

The divergence between radiological success and patient-reported utility requires a mechanistic explanation. Structural integrity does not guarantee optimal range of motion, strength, or patient satisfaction because postoperative function depends on the entire shoulder kinetic chain rather than the tendon-bone interface alone. Early structural failure often occurs within the first postoperative months in large and massive tears (47), but some retears remain clinically tolerated because the deltoid, scapular stabilizers, and residual intact cuff segments may compensate for loss of the repaired tendon. Conversely, a healed repair may still produce suboptimal function when stiffness, pain inhibition, poor rehabilitation progression, or chronic muscle degeneration persists.

Crucially, bio-inductive scaffolds primarily address the regenerative deficit at the tendon surface and enthesis by providing a porous template for host-cell infiltration and collagen deposition. They do not directly reverse chronic pathological changes within the muscle belly. Pre-existing fatty infiltration and irreversible atrophy remain major determinants of outcome and may persist despite successful repair (6, 46). This distinction helps explain why scaffold-induced tendon thickening can coexist with modest or neutral between-group PRO differences in some studies.

The biology-function gap should therefore not be interpreted as evidence that structural healing is unimportant. Instead, it suggests that structural healing is necessary but not always sufficient for clinically meaningful recovery. A rigorous evaluation of bio-inductive scaffolds should integrate imaging endpoints, MCID/PASS-based PRO interpretation, muscle quality, rehabilitation milestones, return-to-work outcomes, and cost-effectiveness. Such an approach aligns the biological mechanism of the implant with the patient-centered outcomes that ultimately determine clinical value.

## 7 Safety profile

The introduction of any xenograft material into a synovial joint inherently requires careful evaluation for infection, immune-mediated synovitis, foreign-body reaction, inflammatory stiffness, implant migration, and fixation-related complications. However, the currently available clinical evidence suggests that modern highly porous bovine collagen scaffolds have a generally favorable short- to mid-term safety profile when implanted using appropriate technique and postoperative protocols. In the Level I randomized trial by Ruiz Ibán et al. (39), deep infection rates were identical between the scaffold and control groups (1.6%), with no reports of systemic allergic reactions or overt graft rejection. Similarly, recent randomized and prospective clinical studies have reported low rates of serious implant-related adverse events while demonstrating favorable structural healing profiles (15, 33, 40). These findings support the biocompatibility of purified collagen-based matrices, although they should be interpreted with the understanding that most studies remain underpowered to detect rare immune or infectious events (17, 37).

Early and intermediate clinical studies in partial- and full-thickness tears provide additional reassurance regarding local tolerability. Prospective imaging-based series have shown progressive implant incorporation, increased tendon thickness, and the absence of a consistent signal for severe xenograft rejection or destructive synovitis (25, 26). Histological and systematic-review evidence also indicates that the implant is gradually replaced by host-derived collagenous tissue rather than persisting as a permanent foreign body (17, 25). Nevertheless, the absence of frequent catastrophic complications should not be equated with complete risk elimination. Rare or technique-sensitive problems, including implant prominence, edge lift-off, staple pull-out, subacromial abrasion, excessive local inflammatory response, and postoperative stiffness, remain clinically relevant considerations, particularly during the surgeon’s learning curve (34, 48).

Postoperative stiffness deserves specific attention because it represents the most plausible safety signal at the interface between biological induction and rehabilitation. The editorial concern raised by Tansey and Lindeman emphasizes that collagen patch augmentation appears to increase tendon thickness with relatively few reported complications, but that indications, efficacy, and stiffness risk require careful interpretation (48). Local cellular infiltration and matrix deposition are expected components of bio-induction; however, if coupled with prolonged immobilization, excessive pain inhibition, or aggressive inflammatory response, this biological activity may contribute to transient range-of-motion limitation. Therefore, postoperative rehabilitation should be individualized: early controlled motion may reduce adhesions and stiffness, but the scaffold should not be treated as a time-zero load-bearing graft capable of permitting unrestricted early loading (24, 32, 48).

Overall, the safety profile of bio-inductive collagen scaffolds appears acceptable, but not definitively established for all patient phenotypes. Current evidence supports low observed rates of infection, systemic immune reaction, and graft rejection in controlled and prospective cohorts (15, 17, 33, 39, 40). At the same time, the literature is limited by heterogeneous reporting of adverse events, variable definitions of stiffness, relatively short follow-up, and limited power for rare complications. Accordingly, safety should be framed as conditional rather than absolute: these implants are best used with meticulous placement, avoidance of implant prominence, careful staple deployment, standardized complication surveillance, and rehabilitation protocols that balance early mobility with protection of the underlying repair (34, 48).

## 8 Value, alternatives, and the selection dilemma

### 8.1 The economic battlefield: societal vs. payor perspectives

In an era defined by cost containment and value-based care, the routine incorporation of commercial bio-inductive implants, which may add substantial device-related expense to each procedure, requires more than a demonstration of improved imaging-based healing. Economic value should be evaluated across multiple domains, including implant acquisition cost, operative time, revision surgery avoidance, rehabilitation burden, time away from work, and quality-adjusted life-year gain. This broader framework is important because rotator cuff repair itself has been shown to generate substantial societal value when successful healing restores function and reduces indirect productivity losses (49). Therefore, the economic question is not simply whether a scaffold is expensive, but whether its incremental cost is offset by a sufficiently large reduction in structural failure, faster recovery, or avoidance of revision surgery in the subgroup being treated.

From a societal perspective, bio-inductive scaffolds may be more defensible when indirect costs are incorporated. Longo et al. (17) emphasized that improvements in healing, return-to-work trajectory, and reduced downstream resource use may shift the apparent value of bio-inductive implants when productivity loss is included in the calculation. This logic is particularly relevant for working-age patients, labor-intensive occupations, revision-risk phenotypes, and patients in whom a retear would carry major socioeconomic consequences. However, from a strict hospital or commercial payor perspective, the calculus remains more stringent. McIntyre et al. (50) reported that a resorbable bioinductive collagen implant can be cost-effective in rotator cuff repair, but the model is highly sensitive to implant cost, baseline retear risk, the magnitude of retear reduction, and assumptions regarding downstream costs. Thus, cost-effectiveness is unlikely to be uniform across all rotator cuff tears; it is most plausible in high-risk phenotypes where the absolute reduction in retear risk is large enough to justify the incremental implant expenditure (37–40, 50).

This distinction explains why indiscriminate use in biologically low-risk tears may represent low-value care even when the technology is biologically active. In low-risk small-to-medium tears with good tendon quality, minimal retraction, preserved muscle quality, and a high probability of healing with standard repair, the absolute risk reduction achievable with a costly implant may be small. By contrast, in high-risk tears, revision settings, poor tendon quality, or patient profiles with high socioeconomic consequences from delayed recovery, the same device may offer a more favorable value proposition. Accordingly, economic evaluation should be linked directly to phenotype-specific retear risk rather than applied as a uniform judgment for or against bio-inductive technology (5, 9, 37–40, 44–46, 49, 50).

### 8.2 The autologous renaissance: a “free” gold standard?

Despite the potential for societal cost savings, hospitals operating under fixed budgets face a pragmatic question: why add a commercial implant when the patient may possess a viable autologous graft source? The long head of the biceps tendon (LHB), once treated primarily as a pain generator requiring tenotomy or tenodesis, has increasingly been repurposed as an autologous biological and mechanical augmentation option in large, massive, or irreparable tears. Baumann et al. (51) reported in a meta-analysis that partial repair with biceps augmentation was associated with lower retear rates in large-to-massive tears than partial repair alone. This concept has been further supported by recent clinical, systematic, and biomechanical evidence suggesting that LHB augmentation or biceps-based reconstruction may improve tendon coverage, contribute to superior stability, and provide an economical local tissue option in challenging tears (51–53).

The appeal of autologous tissue is not only economic. Unlike off-the-shelf xenograft or allograft materials, LHB and fascia lata grafts provide living or patient-derived collagenous tissue without immunogenic graft-processing concerns and without direct implant cost. Recent comparative evidence also challenges the assumption that commercial structural patches necessarily outperform autologous alternatives. Kim and Shin compared human dermal allograft patch augmentation with anterior cable reconstruction using biceps tendon autograft in large retracted anterior rotator cuff tears and reported no significant difference in clinical outcomes or tendon integrity between the two approaches (54). Similarly, technical evolution continues to expand autologous options: Jiang et al. (55) described a side-to-side triple-row repair combined with biceps tendon augmentation for massive rotator cuff tears, reporting high short-term healing rates.

When the biceps tendon is absent, unsuitable, or previously treated, fascia lata autograft remains an important biological alternative, particularly in superior capsular reconstruction or medialized repair strategies for large, massive, or irreparable tears. Clinical evidence from fascia lata augmentation studies has demonstrated favorable outcomes in selected large and massive tears (56), while foundational and editorial work in superior capsular reconstruction continues to emphasize the biomechanical and biological advantages of fascia lata autograft in selected irreparable patterns (57, 58). Nevertheless, autologous options should not be romanticized as truly “free.” They may increase operative complexity, require harvesting or graft preparation, create donor-site morbidity, alter biceps-related function or cosmesis, and may not be available in revision cases or poor-tissue phenotypes. Thus, the economic advantage of autologous augmentation must be balanced against technical feasibility, donor-tissue quality, patient-specific morbidity, and surgeon experience (51–58). The biological, technical, safety, and economic trade-offs between commercial bio-inductive scaffolds and autologous alternatives are summarized in Table 2.

**Table 2:** Comparative analysis of bio-inductive scaffolds and autologous alternatives in rotator cuff repair: mechanism, clinical utility, safety, and value-based selection.

Feature	Bio-inductive scaffold (e.g., Bovine Collagen Implant)	Autologous alternatives (e.g., long head of biceps, fascia lata)
Direct cost and resource implication	Adds a dedicated commercial implant cost; value depends on whether reduced retear, revision avoidance, faster rehabilitation, or return-to-work gains offset the upfront expenditure (49, 50).	No commercial implant acquisition cost, but may increase operative time or require tissue harvest/preparation; cost advantage is greatest when suitable autologous tissue is available and local reimbursement is constrained (51, 53–58).
Primary biological / mechanical mechanism	Biological induction: a porous, temporary extracellular-matrix template that supports host-cell infiltration, angiogenesis, collagen deposition, and tendon-like tissue maturation; it provides limited time-zero load-bearing strength (15, 17, 24–26, 33).	Structural replacement or reinforcement: living or autologous tissue can provide immediate load sharing, cable reconstruction, or gap bridging while avoiding xenograft-related immunogenicity concerns (51, 53–58).
Evidence-supported structural effect	Associated with increased tendon thickness, improved MRI healing, and reduced structural failure or medial-row failure in selected cohorts and comparative studies (15, 17, 27, 33, 37–41).	LHB and fascia lata augmentation have shown favorable structural outcomes in large, massive, or irreparable tears, particularly when native tissue can be repurposed as a load-sharing graft (51, 53–58).
Surgical efficiency and technical demand	Off-the-shelf, low-profile, and relatively rapid to deploy using staple-based fixation; however, success depends on correct bursal-sided positioning, avoidance of folding, and secure fixation (25, 26, 32–36, 48).	Requires tendon preservation/transposition or fascia harvest and graft preparation; technically more demanding, but does not require a commercial scaffold and may be integrated into reconstruction strategies (53–58).
Safety / morbidity profile	Generally acceptable safety profile in reported studies, with no strong signal of deep infection, systemic allergic reaction, or overt graft rejection; rare concerns include stiffness, implant prominence, staple-related irritation, or subacromial abrasion (25, 26, 39, 48).	Avoids xenograft exposure but introduces donor-site or tissue-specific morbidity, such as biceps cramping/cosmesis or fascia harvest morbidity; risks depend on graft source and technique (53–58).
Best-fit indication	Selected repairable tears with compromised biology: poor tendon quality, biologically high-risk healing profile, revision setting, selected small/medium tears with intact rotator cable and high recovery demands, or large/massive tears where biological induction rather than gap bridging is the goal (6, 9, 15, 17, 27, 33, 37–47, 49, 50).	Large, massive, or irreparable tears requiring structural support, anterior cable reconstruction, partial repair reinforcement, or cost-conscious augmentation when LHB or fascia lata is available and biologically suitable (51, 53–58).
Key limitation	High acquisition cost, limited time-zero mechanical reinforcement, and persistent biology-function gap: structural improvement may not exceed MCID/PASS/SCB thresholds in all cohorts and cannot reverse severe fatty infiltration or muscle atrophy (6, 9, 34, 37–39, 42–47, 49, 50).	Availability and quality of autologous tissue are variable; harvest or transposition may add morbidity and operative complexity, and high-quality direct comparative evidence remains limited for some techniques (53–58).
Value-based verdict	Most defensible as a selective, phenotype-specific intervention when predicted structural failure risk, tissue biology, patient demand, or return-to-work priority justifies the cost; not supported as indiscriminate routine augmentation for all tears (9, 15, 17, 27, 33, 37–41, 49, 50, 59).	Represents a cost-conscious and biologically familiar option when structural tissue is required and suitable autologous tissue is available; may serve as a pragmatic first-line augmentation strategy in resource-sensitive settings (51, 53–58).

### 8.3 Stratified application

Given the heterogeneity of rotator cuff tear morphology and patient-specific biological risk, the use of bio-inductive scaffolds should be guided by a stratified, phenotype-specific framework rather than by tear size alone. Current evidence and the 2025 AAOS Clinical Practice Guideline do not support a blanket recommendation against bio-inductive implants in small-to-medium tears; instead, the guideline supports bioinductive tendon implants as an augmentation strategy or alternative to non-augmented repair when appropriately indicated (9). This position is reinforced by the randomized controlled trial by Camacho Chacón et al. (40), in which isolated bioinductive repair for selected small/medium full-thickness supraspinatus tears with an intact rotator cable resulted in improved tendon quality, increased tendon thickness, complete MRI healing at 12 and 24 months, superior patient-reported outcomes, and faster return to work compared with sutured repair. Therefore, small-to-medium tear size should not be treated as an exclusion criterion for bio-inductive technology.

Instead, the relevant distinction is between low-risk tears with high intrinsic healing potential and biologically or functionally high-risk lesions in which augmentation may provide meaningful structural, clinical, or recovery-related value. Established prognostic factors for healing include tear size, tendon retraction, tendon quality, fatty infiltration, muscle atrophy, patient age, bone mineral density, metabolic risk factors, smoking, and work or activity demands (1, 5, 9, 44–46). In truly low-risk tears, characterized by robust tendon quality, minimal retraction, preserved muscle quality, low functional demand, and a high expected probability of healing with standard arthroscopic repair, the routine addition of a high-cost implant remains a value-sensitive decision (5, 34, 37, 38, 50). Conversely, bio-inductive scaffolds may be justified in selected small-to-medium tears when poor tendon quality, intact rotator cable morphology suitable for isolated bioinductive repair, biological risk factors, early return-to-work needs, or revision circumstances increase the expected value of biological induction (9, 40, 44–46).

For large-to-massive tears, where the baseline risk of structural failure is substantially higher, the rationale for biological or structural augmentation is stronger, but the choice of augmentation should remain individualized. Bio-inductive scaffolds may be favored when the primary objective is host-tissue induction, tendon thickening, and low-profile biological reinforcement rather than mechanical gap bridging (15, 17, 33, 37–40). Structural patches or autologous grafts may be more appropriate when a mechanical defect requires load sharing, when tendon excursion is insufficient, or when superior capsular reconstruction is needed (27, 54, 56–58). When suitable tissue is available, LHB or fascia lata grafts may provide cost-conscious alternatives to commercial implants, particularly in health systems where direct implant cost drives access and reimbursement (51–58). In cases of terminal muscle degeneration, severe fatty infiltration, advanced irreparability, or pseudoparalysis, however, neither bio-inductive scaffolds nor autologous augmentation can reverse established muscle pathology, and reconstructive or salvage procedures should be considered instead (5, 9, 44–46, 57, 58).

Thus, the proposed stratification should be interpreted as a precision-medicine and value-based framework, not as a recommendation against bio-inductive augmentation in small-to-medium tears. The clinical question is not whether bio-inductive implants are useful in principle, but which tear phenotypes and patient profiles are most likely to derive sufficient structural, functional, recovery-related, or socioeconomic benefit to justify their use. A practical decision algorithm should therefore integrate tear size, rotator cable integrity, tendon quality, fatty infiltration, retraction, age, comorbidity burden, occupational demand, revision risk, availability of autologous tissue, and local cost constraints (1, 5, 9, 37–40, 44–46, 49–58).

## 9 Future directions: the AI frontier and material evolution

### 9.1 Predictive algorithms as clinical gatekeepers

To date, the decision to use biological augmentation has often relied on intraoperative surgeon judgment, including subjective impressions of tendon quality, tissue mobility, tear chronicity, and repair tension. Although such experience remains indispensable, a purely intuition-based approach is vulnerable to inter-surgeon variability and may contribute to inconsistent health-economic deployment of high-cost implants. A more rigorous future framework should therefore integrate established clinical predictors of failed healing, including age, tear size, tendon retraction, fatty infiltration, bone mineral density, metabolic comorbidities, and occupational demand, into reproducible preoperative risk models (5, 6, 9, 27–30, 42–47).

Artificial intelligence (AI), radiomics, and deep learning may provide the technical infrastructure for this transition. Rather than simply classifying the presence or absence of a tear, next-generation models should quantify the biological and mechanical features that determine whether augmentation is likely to add value. Preoperative MRI contains information on tendon stump quality, muscle fatty infiltration, muscle atrophy, footprint bone quality, and tear morphology that may be difficult to integrate consistently by visual inspection alone. Huo et al. (59) recently reported that a deep-learning model could predict retear after arthroscopic rotator cuff repair with high accuracy using preoperative imaging and clinical data, illustrating the potential of AI-based risk stratification in this field.

In practical terms, the most clinically useful application would not be a stand-alone algorithm that replaces surgeon judgment, but a decision-support tool embedded in the Picture Archiving and Communication System (PACS) or electronic medical record. Such a system could generate a calibrated “Retear Risk Probability Score” before surgery and stratify patients into low-, intermediate-, and high-risk categories. Low-risk patients with favorable tendon quality and low predicted retear probability could be treated with standard repair, thereby avoiding unnecessary implant cost. Conversely, patients with high predicted structural risk could be considered for bio-inductive scaffolds, dermal allografts, or autologous augmentation, depending on tear morphology, tissue availability, and cost context (34, 37, 39, 40, 49, 50, 52–58). This approach aligns the use of expensive biologics with the subgroup most likely to benefit from structural reinforcement, rather than applying augmentation indiscriminately.

However, enthusiasm for AI should be tempered by important methodological limitations. Many existing models are derived from single-center retrospective datasets and may be vulnerable to spectrum bias, scanner-specific imaging features, inconsistent MRI protocols, and limited external validation. Before AI can function as a true “precision gatekeeper,” future studies must demonstrate prospective multicenter validity, transparent model calibration, clinically interpretable outputs, and evidence that model-guided augmentation actually improves patient outcomes or cost-effectiveness compared with standard surgeon-directed decision-making (59).

### 9.2 Next-generation materials: gradient, bioactive, and 4D-responsive scaffolds

While predictive algorithms refine who should receive augmentation, material science is redefining what the implant should accomplish. Current commercial bio-inductive collagen scaffolds primarily increase tendon thickness by serving as porous templates for host-cell infiltration and collagen deposition. This strategy is biologically useful, but it does not fully recapitulate the native tendon-to-bone enthesis, which contains spatial gradients in collagen alignment, mineral content, cellular phenotype, and elastic modulus. Consequently, future scaffold development is shifting from homogeneous collagen sheets toward biomimetic, multiphasic, and spatially graded constructs that more closely reproduce the tendon-fibrocartilage-bone transition (60–64).

Several design principles are particularly relevant to rotator cuff repair. First, scaffold architecture should reproduce anisotropic tendon mechanics by guiding collagen alignment along the native direction of load. Second, the bone-facing region should promote mineralization and fibrocartilage formation, while the tendon-facing region should support tenogenic matrix organization. Third, bioactive delivery systems should provide temporally controlled release of growth factors, stem-cell-derived signals, or immunomodulatory cues rather than relying on a single early biological stimulus. Foundational interface-engineering studies have shown that nanofiber-based and triphasic scaffolds can be designed to reproduce aspects of the tendon-to-bone transition, providing a mechanistic template for future rotator cuff-specific materials (61–64).

In this context, gel-based composite scaffolds, high-resolution 3D bioprinting, and 4D-responsive materials offer a plausible next step. As discussed by Tharakan et al. (60), gel-based composite systems may allow spatial tuning of mechanical stiffness, porosity, bioactive molecule distribution, and cellular microenvironment within the same implant. Unlike static mono-component scaffolds, 3D-printed or 4D-responsive constructs could theoretically adapt to local mechanical loading, hydration state, or degradation kinetics, thereby narrowing the mismatch between soft tendon and rigid bone. These technologies remain largely preclinical, and their translation will require demonstration of manufacturability, sterilization stability, mechanical durability, biocompatibility, and superiority over existing collagen implants in clinically relevant large-animal or human studies.

**Figure 3:**
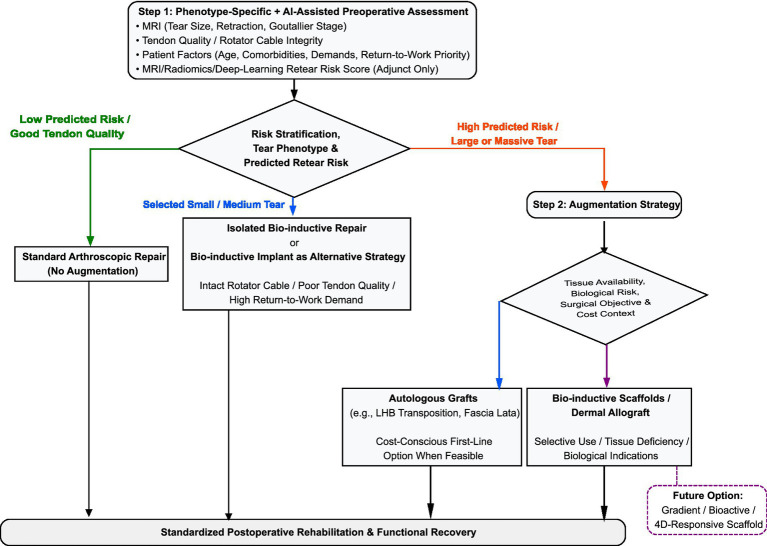
Proposed phenotype-specific and AI-assisted decision algorithm for biological augmentation in rotator cuff repair. Comprehensive preoperative assessment should integrate tear morphology, tendon quality, rotator cable integrity, retraction, fatty infiltration, muscle atrophy, patient age, bone quality, comorbidities, revision status, functional demands, and cost constraints (1, 5, 6, 9, 42–47, 49, 50, 52–58). When available, MRI-based radiomics or deep-learning models may provide an individualized Retear Risk Probability Score, but these tools should complement rather than replace surgeon judgment and require prospective validation (59). Low-risk tears with good tendon quality and low predicted retear risk may be treated with standard arthroscopic repair without augmentation. Selected small-to-medium tears with intact rotator cable integrity, compromised tendon quality, biological risk factors, or high return-to-work demands may be candidates for isolated bio-inductive repair or bio-inductive implants as an alternative strategy (9, 40). In high-risk large or massive tears, augmentation should be individualized according to tissue availability, predicted failure risk, surgical objective, and cost context (15, 17, 27, 33, 37–41, 49, 50, 52–58). Autologous grafts, such as long head of the biceps or fascia lata, may be preferred when feasible, whereas bio-inductive scaffolds or dermal allografts should be reserved for selective biological indications or tissue deficiency (49, 50, 52–58). Future gradient, bioactive, or 4D-responsive scaffolds may further refine this algorithm by better reproducing the native tendon-to-bone transition (60–64). All pathways should be followed by standardized postoperative rehabilitation.

Taken together, the next generation of rotator cuff augmentation will likely depend on the convergence of predictive analytics and biomaterial engineering. AI-driven risk stratification may identify the patients most likely to benefit, whereas graded and bioactive scaffolds may provide a more anatomically faithful solution for restoring the tendon-to-bone interface. The central challenge for future research is to link these innovations to measurable clinical value: lower retear rates, faster recovery, improved patient-reported outcomes, fewer revisions, and acceptable cost-effectiveness. Together, AI-assisted risk stratification and next-generation scaffold design may support a more individualized decision algorithm for biological augmentation in rotator cuff repair (Figure 3).

## 10 Conclusion

Bio-inductive scaffolds represent a biologically plausible and increasingly evidence-supported adjunct for rotator cuff repair, particularly in high-risk tear phenotypes, revision settings, poor tendon quality, and patients with biological or functional risk factors that compromise healing. Recent meta-analyses, randomized trials, prospective multicenter studies, and imaging-based cohort data consistently support their capacity to improve structural integrity by increasing tendon thickness and reducing retear rates, including medial-row or type 2 failure patterns (15, 17, 27, 33, 37–41). However, structural success should not be interpreted as synonymous with clinical utility. Several comparative studies have shown that improvements in anatomical healing may be accompanied by small, statistically non-significant, or clinically subthreshold differences in ASES, Constant-Murley, UCLA, or other patient-reported outcomes, particularly when assessed against MCID, PASS, or substantial clinical benefit thresholds (6, 34, 37–39, 42, 43).

This biology-function gap underscores that postoperative recovery after rotator cuff repair is multifactorial. Tendon continuity is important, but clinical function is also shaped by tear chronicity, tendon retraction, rotator cable integrity, fatty infiltration, muscle atrophy, bone quality, age, metabolic comorbidities, rehabilitation quality, occupational demands, and patient expectations (5, 9, 27–30, 44–47). Accordingly, bio-inductive implants should be viewed as biological tools that may improve the local healing environment, rather than as universal solutions capable of reversing established muscle degeneration or guaranteeing superior patient-perceived outcomes.

Importantly, our value-based caution should not be interpreted as a recommendation against bio-inductive implants in small-to-medium tears. The 2025 AAOS Clinical Practice Guideline and recent randomized evidence support their selective use in appropriately indicated small/medium tears, particularly when rotator cable integrity, tendon quality, patient-specific biological risk, or recovery demands favor a bio-inductive strategy (9, 40). The central issue is therefore not whether bio-inductive scaffolds can be effective, but which tear phenotypes and patient profiles are most likely to derive sufficient structural, functional, or recovery-related benefit to justify their use.

Future research should move beyond binary comparisons of scaffold versus no scaffold and instead refine phenotype-specific indications, standardized imaging endpoints, patient-centered outcome thresholds, cost-effectiveness models, and head-to-head comparisons with autologous alternatives such as long head of the biceps or fascia lata grafts (49, 50, 52–58). In parallel, externally validated AI or radiomics-based risk models may help identify patients with a sufficiently high predicted retear risk to warrant augmentation, while next-generation gradient, bioactive, or 4D-responsive scaffolds may better reproduce the native tendon-to-bone transition (59–64). Such an evidence-based, precision-medicine framework will be essential to optimize both clinical efficacy and societal healthcare value.
